# Impact of microvascular invasion risk on tumor progression of hepatocellular carcinoma after conventional transarterial chemoembolization

**DOI:** 10.1093/oncolo/oyae286

**Published:** 2024-10-30

**Authors:** Guanhua Yang, Yuxin Chen, Minglei Wang, Hongfang Wang, Yong Chen

**Affiliations:** The First School of Clinical Medicine, General Hospital of Ningxia Medical University, Yinchuan, People’s Republic of China; Department of Paediatrics, Division of Respiratory Medicine and Allergology, Sophia Children’s Hospital, Erasmus MC, Rotterdam, The Netherlands; Department of Radiology and Nuclear Medicine, Erasmus MC, Rotterdam, The Netherlands; The First School of Clinical Medicine, General Hospital of Ningxia Medical University, Yinchuan, People’s Republic of China; The First School of Clinical Medicine, General Hospital of Ningxia Medical University, Yinchuan, People’s Republic of China; Department of Interventional Radiology, General Hospital of Ningxia Medical University, Yinchuan, People’s Republic of China

**Keywords:** hepatocellular carcinoma, transarterial chemoembolization, microvascular invasion, prognosis

## Abstract

**Objective:**

To assess tumor progression in patients with hepatocellular carcinoma (HCC) without macrovascular invasion who underwent treatment with conventional transarterial chemoembolization (cTACE) based on microvascular invasion (MVI) risk within 2 years.

**Methods:**

This retrospective investigation comprised adult patients with HCC who had either liver resection or cTACE as their first treatment from January 2016 to December 2021. A predictive model for MVI was developed and validated using preoperative clinical and MRI data from patients with HCC treated with liver resection. The MVI predictive model was applied to patients with HCC receiving cTACE, and differences in tumor progression between the MVI high- and low-risk groups were examined throughout 2 years.

**Results:**

The MVI prediction model incorporated nonsmooth margin, intratumoral artery, incomplete or absent tumor capsule, and tumor DWI/T2WI mismatch. The area under the receiver operating characteristic curve (AUC) for the prediction model, in the training cohort, was determined to be 0.904 (95% CI, 0.862-0.946), while in the validation cohort, it was 0.888 (0.782-0.994). Among patients with HCC undergoing cTACE, those classified as high risk for MVI possessed a lower rate of achieving a complete response after the first tumor therapy and a higher risk of tumor progression within 2 years.

**Conclusions:**

The MVI prediction model developed in this study demonstrates a considerable degree of accuracy. Patients at high risk for MVI who underwent cTACE treatment exhibited a higher risk of tumor progression within 2 years.

## Teaser

This study assessed tumor progression in patients with hepatocellular carcinoma without macrovascular invasion who underwent treatment with conventional transarterial chemoembolization based on microvascular invasion risk within 2 years.

**Implications for Practice**

The presence of microvascular invasion (MVI) after surgical resection or liver transplantation is known to be a poor prognostic factor for patients with hepatocellular carcinoma (HCC). This study found that among patients with HCC undergoing conventional transarterial chemoembolization, those classified as high risk for MVI had a lower rate of achieving a complete response after the first tumor therapy and a higher risk of tumor progression within 2 years.

## Introduction

Hepatocellular carcinoma (HCC) is a widespread cancer with aggressive characteristics, demonstrating significant heterogeneity at the genetic, molecular, and histological levels.^1-3^ This intricacy makes it difficult to manage and treat patients with HCC. Microvascular invasion (MVI), a histopathological characteristic of HCC, is correlated with increased invasiveness and is recognized as a significant prognostic indicator for unfavorable consequences in patients who underwent hepatectomy and liver transplantation.^4,5^ As such, prior MVI detection is crucial for personalized treatment plans. Performing an anatomic hepatectomy reduces the likelihood of early recurrence in individuals who test positive for MVI.^2^ MVI-positive patients who had undergone radiofrequency ablation were found to be more vulnerable to recurrence than those who had received hepatectomy.^6^ Previous research has demonstrated that imaging features can preoperatively predict MVI in HCC.^7-10^

Transarterial chemoembolization (TACE) is the main therapy method for individuals diagnosed with intermediate-stage HCC,^1-3^ and it may serve as a viable alternative for individuals with extremely early and early HCC who are unsuitable for hepatectomy, ablation, or transplantation.^11,12^ The heterogeneity of HCC implies that the efficacy of TACE may vary among patients. The response rate following TACE ranges from 15% to 85%, with the majority of patients (70%-80%) succumbing to tumor progression.^13,14^ There has been limited research on the influence of MVI as a biomarker of HCC invasiveness on tumor progression post-TACE.

We proposed creating and verifying a risk prediction model using MRI scans to ascertain the likelihood of MVI in patients with HCC. Additionally, the study intended to analyze tumor progression over a period of 2 years after cTACE treatment based on the anticipated risk of MVI.

## Patients and methods

### Patients

**Figure 1.**
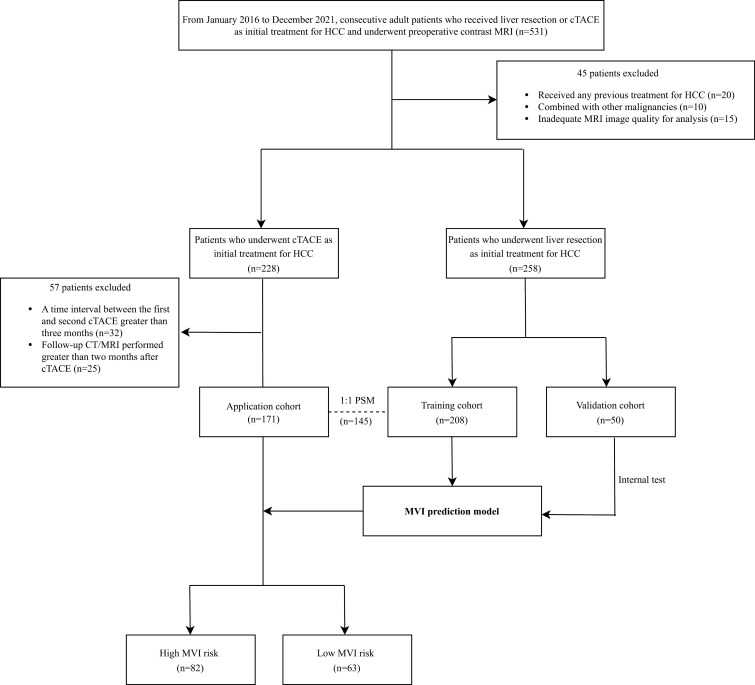
Study flow chart.

This retrospective research (KYLL-20241143) was authorized by the institutional review boards of the General Hospital of Ningxia Medical University. The prerequisites for obtaining informed consent were exempted. This study sequentially included adult patients with HCC who underwent liver resection as their first therapy from January 2016 to December 2021. We proposed creating and verifying a prediction model for MVI. Patients were classified into a training cohort (TC; January 2016-December 2019) and a validation cohort (VC; January 2021-December 2021) based on the time frame in which they were included (Figure 1). Adult patients with HCC who received cTACE as their first therapy between January 2016 and December 2021 were consecutively enrolled and designated as the application cohort to examine the influence of MVI on tumor progression after cTACE.^6,15^

The HCC diagnosis depends on the criteria established by the American Association for the Study of Liver Diseases.^3^ The guidelines for participation in this investigation are as follows: (a) patients aged 18 years or older; (b) patients with liver function categorized as Child-Pugh A or B and Eastern Cooperative Oncology Group performance status (ECOG PS) of 0; (c) patients without macrovascular invasion, extrahepatic metastases, or satellite lesions; (d) patients who have undergone preoperative improved MRI examination within 1 month prior to treatment; (e) complete clinical and follow-up data, in which HCC was pathologically confirmed in all liver resection patients and MVI status were analyzed. Exclusion criteria: patients with (a) any previous therapy for HCC, (b) coupled with other malignancies, and (c) MRI scans of insufficient quality for image analysis. Moreover, the unique inclusion criteria for the cTACE cohort under study included patients meeting the following requirements: (a) to evaluate the tumor’s response using the modified Response Evaluation Criteria in Solid Tumors (mRECIST), it is necessary to have at least one identifiable target lesion^16^; (b) a time interval of <3 months between the initial and subsequent cTACE sessions, with a radiographic complete response (CR) mandated if only one cTACE procedure was conducted; and (c) a time interval of <2 months between follow-up CT or MRI scans post cTACE treatment.

Baseline demographic, clinical, and laboratory data, including age, gender, BCLC stage, Child-Pugh classification, the existence of liver cirrhosis, and pertinent laboratory values (eg, serum alpha-fetoprotein [AFP]), were collected throughout 2 weeks before liver resection or cTACE.

### MRI technique

The MRI tests were conducted using GE Signa 3.0T HD scanners. The MRI scans comprised the subsequent sequences: (a) T2-weighted imaging; (b) diffusion-weighted imaging; (c) in- and opposed-phase T1-weighted imaging; (d) dynamic T1-weighted imaging in the precontrast, early arterial, late arterial, portal venous, and delayed phases. The specific details of the MRI parameters may be found in Supplementary Table S1.

### Image analysis

Two radiologists with 7 and 16 years of experience in liver MRI, conducted the image analysis. They lacked knowledge of any pathological or clinical data. If the 2 radiologists disagreed on imaging characteristics, they discussed it and came to a final consensus. The patients with numerous tumors were assessed based on the tumor with the greatest diameter. The following imaging characteristics were ascertained: (a) the tumor size was ascertained by estimating the maximum distance between the outside edges^17^; (b) the tumor margin was classified into 2 categories: (i) smooth margin, characterized by tumors with a smooth contour, and (ii) nonsmooth margin, characterized by either a noncircumscribed margin with an indistinct transition or small outgrowth nodules protruding into the nontumor parenchyma on transverse and coronal images^8^; (c) tumor growth pattern was classified as (i) intrahepatic growth, and (ii) extrahepatic growth^9^; (d) intratumoral artery was defined as dilated, tortuous, and disordered small vascular developing inside the tumor^8^; (e) peritumoral enhancement was characterized by irregularly enhancing patches detected at the periphery of hepatic tumors throughout the arterial phase, which then transitioned into a isointense signal with the surrounding liver parenchyma throughout the delayed phase^18^; (f) tumor capsules were radiological classified radiologically into 2 categories: complete tumor capsules, which fully encircle the tumor, and incomplete or absent tumor capsules, which only partially enclose the tumor or cannot be identified radiologically^18^; (g) mosaic architecture was characterized by the presence of internally dispersed nodules or compartments within a mass that exhibit varying shapes upon enhancement^17^; (g) intratumoral hemorrhage; (h) intratumoral necrosis; (i) The DWI/T2WI mismatch was characterized by observing a larger lesion diameter on diffusion-weighted imaging compared to T2-weighted imaging in the slices where the lesion diameter was the greatest. The intensity of the mismatch area on DWI was intermediate, being lower than that of the tumor but greater than that in the liver parenchyma^19^; (j) enhancement pattern was classified into 2 categories: (i) typical enhancement, characterized by tumor non-rim-like enhancement in the arterial phase with nonperipheral washout, and (ii) atypical enhancement, characterized by tumor rim-like enhancement in the arterial phase, minimal enhancement, persistent enhancement, and heterogeneous enhancement.^17^

### Histopathological analysis

The gold standard for detecting MVI was established by histopathological examination of surgical specimens collected using a standardized 7-site sampling approach. MVI is defined by the occurrence of cancer cell clusters inside blood vessels coated with endothelial cells, often seen during the invasion of portal vein branches in HCC, including intracapsular blood vessels.^20^

### Predictive model derivation and validation

The clinical and MRI data from the TC were used to identify potential variables and develop MVI nomogram prediction models, while the data from the VC were used to ascertain the predictive accuracy of the model. The research used the consistency index to ascertain the effectiveness of the nomogram in distinguishing between different groups. It also analyzed the calibration and overall benefit of the nomogram in both the TC and VC using calibration and decision curve analysis. The nomogram’s ideal cutoff value was found by doing receiver operating characteristic curve (ROC) analysis and identifying the highest Youden index. This value was subsequently used to classify patients with HCC according to their MVI risk.

### Conventional TACE procedure

The cTACE therapy was carried out by a team of 5 highly skilled interventional radiologists, each with over 5 years of experience. The methodology used followed closely that of previous studies.^21,22^ In conclusion, arteriography was performed by inserting a 5-French catheter into either the celiac trunk or superior mesenteric artery. This was then followed by the precise placement of a 2.7 French microcatheter into the specific artery that supplies blood to the tumor. The nidus was embolized with 20 mg pirarubicin and 10 mL of iodized oil, followed by further embolization with gelatin sponge particles until blood flow in the tumor-feeding artery was halted.

### Tumor response assessment and follow-up

All patients with HCC who underwent cTACE were regularly monitored post-discharge, with follow-up appointments scheduled every 1-2 months for the initial 6 months, followed by checkups every 3-6 months thereafter. Radiographic assessments of primary tumor response were conducted using mRECIST. Patients who did not achieve a CR following the first therapy were evaluated for cTACE retreatment if no contraindications existed. If CR or partial response (PR) was not achieved after 2 cTACE procedures, alternative treatment options were considered and reviewed with the multidisciplinary tumor committee for exclusion from the study cohort. The primary outcome measure of the trial was progression-free survival (PFS), which is the duration from the start of the initial cTACE treatment to the identification of imaging indicative of tumor progression or mortality from any etiology. In cases where tumor progression did not occur, participants were monitored for a minimum of 2 years, with data collection on 31 December 2023. Tumor progression was delineated as the occurrence of local recurrence or advancement of target lesions, emergence of new intrahepatic lesions, the formation of multiple intrahepatic metastases, and the presence of extrahepatic metastases throughout the follow-up period.

### Predictive model application in the cTACE cohort

Tumor progression was assessed in various MVI risk groups within the cTACE cohort using the MVI prediction model. Propensity score matching (PSM) was implemented to decline the impact of selection bias and confounding factors across the TC and application groups. The factors encompassed in the propensity score model were age, gender, hepatitis B virus infection status, cirrhosis status, Child-Pugh classification, and tumor diameter. The propensity score was computed using logistic regression and then paired using the 1:1 closest neighbor matching approach with a caliper value of 0.02.

### Statistical analysis

Continuous variables were ascertained using the Mann-Whitney *U* test (median and interquartile range), while categorical variables were estimated using the χ^2^ or Fisher’s exact tests (number of cases and percentage). In order to find separate risk variables for MVI and create nomograms, univariate and multivariate logistic regression analyses were implemented. The Kaplan-Meier approach was used to assess relapse-free survival curves, and the log-rank test was used to investigate subgroup variations. SPSS software (version 26.0) and R software (version 3.6.1) were used to conduct statistical analyses. The significance level was set at *P* < .05, and all statistical tests were conducted using 2-tailed tests.

## Results

### Baseline patient characteristics

The investigation included a cohort of 429 patients, consisting of 336 males (78.3%) and 93 females (21.7%) with a median age of 56 years (range 42-65). All patients’ fundamental features are presented in Table 1 and Supplementary Tables S2 and S3. Histopathological study revealed MVI in 128 (61.5%) and 30 (60.0%) patients in the TC and VC, respectively. There were no significant variations in demographic and clinicopathological factors between the 2 groups (*P* > .05).

**Table 1.** Baseline characteristics of patients undergoing liver resection.

Variable	All patients (*n* = 258)	Training cohort (*n* = 208)	Validation cohort (*n* = 50)	*P* value
Age (years)^a^	56 (48-64)	55 (48-65)	56 (48-62)	.776
Sex				
Male	196 (76.0)	159 (76.4)	37 (74.0)	.717
Female	62 (24.0)	49 (23.6)	13 (26.0)	
Hepatitis B virus Infection				
Absent	36 (14.0)	27 (13.0)	9 (18.0)	.358
Present	222 (86.0)	181 (87.0)	41 (82.0)	
Cirrhosis				
Absent	78 (30.2)	60 (28.8)	18 (36.0)	.323
Present	180 (69.8)	148 (71.2)	32 (64.0)	
Child-Pugh				
A	237 (91.9)	190 (91.3)	47 (94.0)	.538
B	21 (8.1)	18 (8.7)	3 (6.0)	
BCLC stage				
0	23 (8.9)	20 (9.6)	3 (6.0)	.693
A	222 (86.0)	178 (85.6)	44 (88.0)	
B	13 (5.1)	10 (4.8)	3 (6.0)	
Serum creatinine (μmol/L)^a^	64.8 (55.4-72.4)	64.0 (55.0-71.8)	66.2 (56.7-74.7)	.284
Serum total bilirubin (μmol/L)^a^	16.1 (12.4-21.6)	16.1 (12.3-21.7)	16.3 (13.7-20.7)	.807
Serum albumin (g/L)^a^	38.9 (35.3-41.4)	38.7 (35.2-41.3)	39.2 (36.4-41.8)	.351
Prothrombin time (s)^a^	12.3 (11.6-13.3)	12.3 (11.6-13.4)	12.4 (11.5-13.1)	.999
International normalized ratio^a^	1.1 (1.0-1.1)	1.1 (1.0-1.1)	1.1 (1.0-1.1)	.955
AST (U/L)^a^	32.6 (24.2-45.3)	32.6 (24.2-45.0)	32.0 (25.1-50.6)	.869
ALT (U/L)^a^	34.7 (24.9-48.8)	35.6 (24.6-52.0)	29.9 (24.7-40.5)	.130
Neutrophils count (× 10^9^/L)^a^	2.8 (2.1-3.7)	2.8 (2.1-3.8)	2.9 (2.3-3.5)	.632
lymphocyte count (× 10^9^/L)^a^	1.6 (1.2-2.0)	1.6 (1.2-2.0)	1.5 (1.1-1.9)	.271
Platelet count (× 10^9^/L)^a^	148.0 (109.0-199.3)	146.5 (109.0-195.8)	160.5 (111.0-211.0)	.321
AFP (μg/L)^a^	75.7 (7.0-1210.0)	75.8 (6.5-1210.0)	72.2 (8.2-989.3)	.994
Tumor diameter (cm)^a^	5.3 (3.0-7.8)	4.9 (2.9-7.8)	5.8 (4.5-8.0)	.072
Tumor number				
1	245 (95.0)	198 (95.2)	47 (94.0)	.729
≥2	13 (5.0)	10 (4.8)	3 (6.0)	
MVI				
Positive	158 (61.2)	128 (61.5)	30 (60.0)	.841
Negative	100 (38.8)	80 (38.5)	20 (40.0)	

Unless otherwise specified, the data are the number of patients, with percentages in parentheses.

^a^The data are presented as median, with interquartile range in parentheses.

Abbreviations: AFP, alpha-fetoprotein; ALT, alanine aminotransferase; AST, aspartate aminotransferase; BCLC, Barcelona Clinic Liver Cancer; MVI, microvascular invasion.

### Development and validation of the nomogram

Table 2 presents a succinct summary of the outcomes from both univariate and multivariate analyses of clinical and MRI features that are associated with MVI positivity in the TC. The multivariate logistic regression analysis was conducted via a backward stepwise selection method, and variables with a significance level (*P* value) of <.05 in the univariate logistic regression were comprised. The multivariate analysis discovered several significant separate predictors of MVI positivity. These predictors include nonsmooth margin (odds ratio [OR] = 0.367; 95% CI [CI] = 0.150-0.896; *P* = .028), intratumoral artery (OR = 0.318; 95% CI = 0.138-0.735; *P* = .007), incomplete or absent tumor capsule (OR = 0.113; 95% CI = 0.047-0.274; *P* < .001), and tumor DWI/T2WI mismatch (OR = 0.125; 95% CI = 0.035-0.449; *P* = .001).

**Table 2.** Predictors for microvascular invasion in the training cohort.

Predictors	Univariable analysis		Multivariable analysis		
	Odds ratio (95% CI)	*P* value	Regression coefficient	Odds ratio (95% CI)	*P* value
Clinical variables					
Age (years)	0.992 (0.967-1.018)	.544			
Sex (male vs female)	1.774 (0.885-3.557)	.106			
Hepatitis B virus Infection (absent vs present)	1.291 (0.550-3.032)	.558			
Cirrhosis (absent vs present)	1.364 (0.727-2.558)	.334			
Serum creatinine (umol/L)	1.003 (0.994-1.011)	.556			
Serum total bilirubin (umol/L)	1.009 (0.988-1.030)	.416			
Serum albumin (g/L)	1.027 (0.967-1.089)	.388			
Prothrombin time (s)	0.802 (0.654-0.982)	.033			
Aspartate aminotransferase (U/L)	1.019 (1.004-1.035)	.013			
Alanine aminotransferas (U/L)	1.001 (0.993-1.010)	.785			
NLR	1.632 (1.209-2.201)	.001			
PLR	1.012 (1.006-1.019)	<.001			
AFP (≤400 vs >400 ng/mL)	0.651 (0.357-1.187)	.161			
Imaging variables					
Tumor diameter (cm)	1.541 (1.339-1.773)	<.001			
Tumor number (1 vs ≥2)	1.489 (0.373-5.916)	.575			
Tumor margin (smooth vs nonsmooth)	0.111 (0.058-0.212)	<.001	1.003	0.367 (0.150-0.896)	.028
Tumor growth pattern (Intrahepatic growth vs extrahepatic growth)	0.403 (0.220-0.740)	.003			
Intratumoral artery (absent vs present)	0.151 (0.079-0.289)	<.001	1.145	0.318 (0.138-0.735)	.007
Peritumoral enhancement (absent vs present)	0.191 (0.088-0.417)	<.001			
Tumor capsule (complete vs incomplete or absent)	0.049 (0.024-0.101)	<.001	2.176	0.113 (0.047-0.274)	<.001
Mosaic architecture (absent vs present)	0.163 (0.088-0.301)	<.001			
Intratumoral hemorrhage (absent vs present)	0.350 (0.158-0.777)	.010			
Intratumoral necrosis (absent vs present)	0.162 (0.086-0.304)	<.001			
DWI/T2WI mismatch (absent vs present)	0.085 (0.029-0.247)	<.001	2.080	0.125 (0.035-0.449)	.001
Typical enhancement (absent vs present)	4.021 (1.940-8.333)	<.001			

Abbreviations: AFP, serum alpha-fetoprotein; NLR, [neutrophils count (×10^9^/L)/lymphocyte count (×10^9^/L)]; PLR, [platelet count (×10^9^/L)/lymphocyte count (×10^9^/L)].

**Figure 2.**
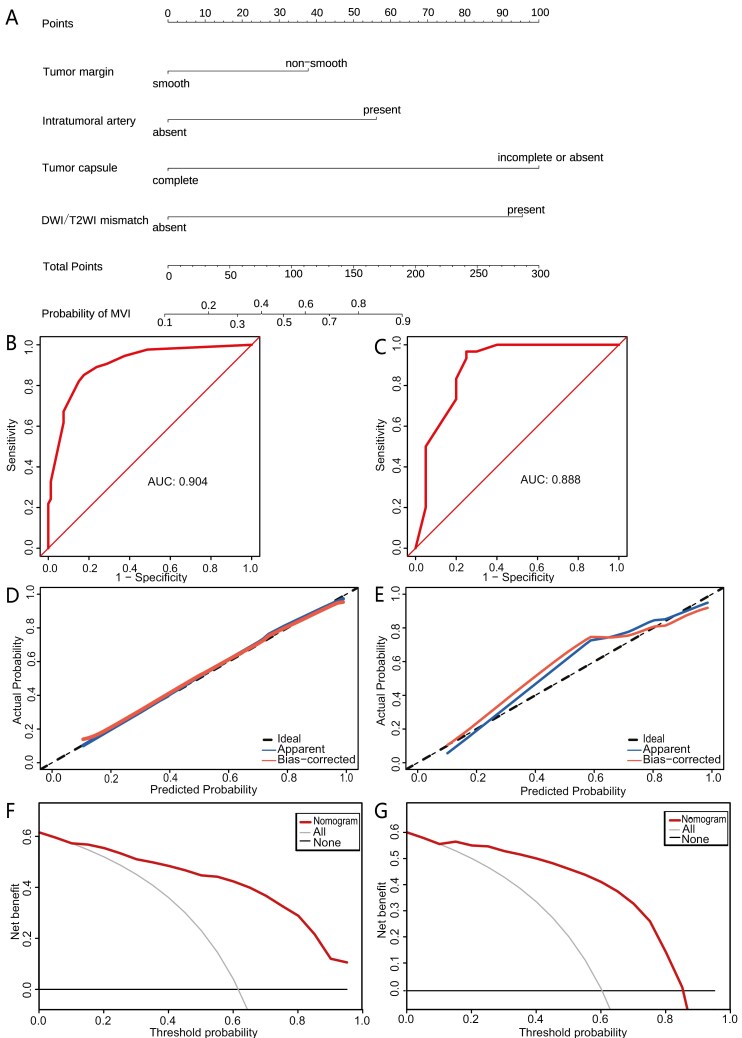
Nomogram for preoperative estimation of microvascular invasion (MVI) risk and its predictive performance. (A) Nomogram model with tumor margin, intratumoral artery, tumor capsule, and tumor DWI/T2WI mismatch. (B, C) Receiver operating characteristic curves of the nomogram in the training and validation cohorts (VCs). (D, E) Calibration curves of the nomogram in the training and VCs. The x-axis represents the nomogram-predicted probability of MVI, the y-axis represents the observed MVI, and the diagonal dashed line signifies the ideal prediction made by a model with perfect accuracy. (F, G) Decision curves of the nomogram in the training and VCs. The gray line represents the net benefit under the assumption that all patients have MVI, while the black line represents the net benefit assuming no patients have MVI. The red line indicates the expected net benefit for the patient as determined by the predictive nomogram.

The nomogram was created using the 4 independent predictors indicated before (Figure 2A). The TC had a consistency index (C-index) of 0.904 (95% CI, 0.862-0.946), while the VC demonstrated a C-index of 0.888 (0.782-0.994), suggesting a strong discriminant performance of the prediction model (Figure 2B and 2C). In the TC, the Hosmer-Lemeshow test yielded a χ^2^ value of 3.847 and a *P* value of .92. In the VC, the test yielded a χ^2^ value of 14.674 and a *P* value of .100. These outcomes manifest that the prediction model fits well. The calibration curves of predicted and observed values had strong concordance in both the TC and VC, as depicted in Figure 2D and 2E. Additionally, the decision curve analysis manifested a greater net benefit than anticipated when encompassing all or no patients expressing MVI (Figure 2F and 2G).

The nomogram’s best cutoff value was determined to be 100 points using the maximum Youden index. This allows for the categorization of patients into 2 groups: those with a low risk of MVI (≤100 points) and those with a high risk of MVI (>100 points). The diagnostic performance markers, encompassing sensitivity, specificity, positive predictive value, and negative predictive value, for diagnosing MVI in the TC were 85.2%, 82.5%, 88.6%, and 77.6%, respectively. These metrics were 96.7%, 75.0%, 85.3%, and 93.8%, respectively, in the VC (Table 3).

**Table 3.** Diagnostic performance of nomogram for predicting MVI-positive HCC.

Variable	Value (95% CI)	
	Training cohort	Validation cohort
Area under ROC curve	0.904 (0.862-0.946)	0.888 (0.782-0.994)
Sensitivity (%)	85.2 (77.8-90.8)	96.7 (82.8-99.9)
Specificity (%)	82.5 (72.4-90.1)	75.0 (50.9-91.3)
Positive predictive value (%)	88.6 (84.1-91.9)	85.3 (75.7-91.5)
Negative predictive value (%)	77.6 (69.5-84.1)	93.8 (68.4-99.0)
Positive likelihood ratio	4.87 (3.0-7.9)	3.87 (1.8 -8.3)
Negative likelihood ratio	0.18 (0.1-0.3)	0.04 (0.006-0.3)

Abbreviations: HCC, Hepatocellular carcinoma; MVI, microvascular invasion; ROC, receiver operating characteristic curve.

### MVI predicts tumor progression risk within 2 years in the cTACE cohort

The cTACE cohort had a median follow-up period of 20.0 (11.0-25.0) months. Table 4 illustrates the advancement of tumors both before and after PSM in the cTACE group. The matching of 145 patients in both the TC and application cohorts after PSM yielded well-balanced baseline characteristics for the 2 groups (Supplementary Table S4). Following PSM in the application cohort, the prediction classified 82 patients (56.6%) as high risk for MVI (Supplementary Figures S1 and S2), while 63 patients (43.4%) were classified as low risk for MVI. Patients classified as high risk for MVI had a poorer initial CR rate (31.7% vs 51.7%, *P* = .002) and a greater occurrence of tumor progression following 2 years (86.6% vs 39.7%, *P* < .001) following cTACE. Among patients with tumor progression, those identified as high risk for MVI exhibited significantly higher incidence of local recurrence or local progression, multiple intrahepatic metastases, and extrahepatic metastases compared with low-risk patients (80.3% vs 68.0%, *P* < .001). In particular, among the subset of patients anticipated to be at greater risk for MVI, 4 out of 5 patients with extrahepatic metastases had multiple metastases in both lungs, while 1 patient had bone metastases in the left 12th rib.

**Table 4.** Analysis of tumor progression within 2 years in the cTACE cohort.

Variable	Before PSM (*n* = 171)			After PSM (*n* = 145)		
	High risk for MVI (*n* = 91)	Low risk for MVI (*n* = 80)	*P* value	High risk for MVI (*n* = 82)	Low risk for MVI (*n* = 63)	*P* value
BCLC stage						
0	12 (13.2)	22 (27.5)	.015	12 (14.6)	17 (27.0)	.110
A	24 (26.4)	26 (32.5)		21 (25.6)	18 (28.6)	
B	55 (60.4)	32 (40.0)		49 (59.8)	28 (44.4)	
Tumor response after initial treatment						
Complete response	28 (30.8)	48 (60.0)	.002	26 (31.7)	36 (57.1)	.020
Partial response	56 (61.5)	29 (36.2)		52 (63.4)	24 (38.1)	
Stable disease	5 (5.5)	2 (2.5)		3 (3.7)	2 (3.2)	
Progressive disease	2 (2.2)	1 (1.3)		1 (1.2)	1 (1.6)	
No tumor progression	12 (13.2)	52 (65.0)	<.001	11 (13.4)	38 (60.3)	<.001
Tumor progression	79 (86.8)	28 (35.0)		71 (86.6)	25 (39.7)	
Local recurrence or local progression	40 (50.6)	16 (57.1)		37 (52.1)	16 (64.0)	
New intrahepatic lesions	16 (20.3)	9 (32.2)		14 (19.7)	8 (32.0)	
Multiple intrahepatic metastases	17 (21.5)	3 (10.7)		15 (21.1)	1 (4.0)	
extrahepatic metastases	6 (7.6)	0 (0)		5 (7.1)	0 (0)	
Time to tumor progression (months), median (range)	12.0 (8.0-18.0)	26.0 (25.0-30.8)	<.001	14.0 (8.8-21.0)	24.0 (15.0-27.0)	<.001

Unless otherwise specified, the data are the number of patients, with percentages in parentheses.

Abbreviations: BCLC, Barcelona Clinic Liver Cancer; cTACE, conventional transarterial chemoembolization; MVI, microvascular invasion; PSM, propensity score matching.

**Figure 3.**
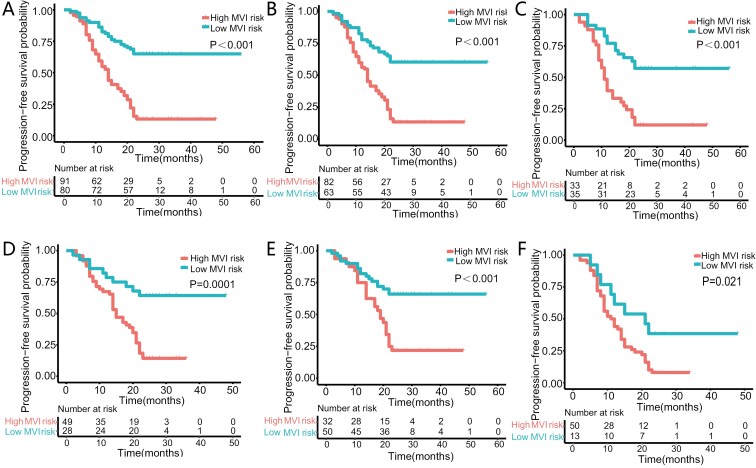
The Kaplan-Meier curves were analyzed for the entire cohort undergoing conventional transarterial chemoembolization (cTACE) prior to propensity score matching (PSM), for the entire cTACE cohort subsequent to PSM, and for the cTACE cohort post-PSM, stratified by Barcelona Clinic Liver Cancer (BCLC) stage (BCLC 0/A and BCLC B) and tumor diameter categories (≤5 cm and >5 cm).

Prior to PSM, patients predicted as high risk for MVI experienced a significantly mitigated median time to tumor development contrasted to patients predicted as low risk for MVI (14 months vs not reached, *P* < .001; Figure 3A). After undergoing PSM, patients categorized as being at high risk for MVI saw a significantly shorter median time to tumor development compared to individuals who were projected to be at low risk (14 months vs not reached, *P* < .001; Figure 3B). In the subgroup analysis of BCLC 0/A patients (*n* = 68) and BCLC B patients (*n* = 77), patients anticipated as high risk for MVI had a significantly mitigated median time to tumor progression compared to those identified as low risk (11 months vs not reached, *P* < .001; 15 months vs not reached, *P* = .0001; Figure 3C and 3D). In the subgroup analysis of patients with tumor diameters of ≤ 5 cm (*n* = 82) and > 5 cm (*n* = 63), patients anticipated as high risk for MVI had a significantly mitigated median time to tumor progression compared to those identified as low risk (19 months vs not reached, *P* < .001; and 11 months vs 21 months, *P* = .021; Figure 3E and 3F).

## Discussion

This investigation created and verified a predictive model for assessing the MVI risk in patients with HCC using preoperative conventional MRI characteristics, demonstrating a high level of discriminatory accuracy. Our findings demonstrate that patients with HCC who are determined to possess a high MVI risk based on the model are more likely to experience accelerated tumor progression within 2 years following cTACE than those anticipated to possess a low MVI risk.

The 4 predictors included in the nomogram are comparable to those identified in previous studies,^6-10^ while discrepancies exist. Of note, the presence of an incomplete or nonexistent tumor imaging “capsule” holds the highest predictive weight within the nomogram, with a score of 100 points, which is consistent with the conclusions drawn by Xu et al^9^ Xu et al^9^ developed a clinical-radiomics model that included 8 predictors with the largest effect on incomplete tumor capsules. Intriguingly, the study found that imaging features (encompassing tumor capsule, tumor margin, and abnormal enhancement around the tumor) were stronger predictors of MVI than radiomics features. The lack of a complete tumor capsule indicates the potential of tumor cells to breach the fibrous tissue barrier and infiltrate the adjacent liver tissue. Furthermore, disrupting intricate vascular channels that connect the tumor interior with the inner and outer layers of the capsule allows tumor cells to enter the vascular lumen, promoting the formation of tumor thrombi.^23,24^

The discrepancy between tumor DWI/T2WI is identified as the second most significant predictor in nomograms, a less commonly used imaging feature in previous research.^7-10,18,19^ Yang et al^19^ first proposed this feature for predicting MVI in patients with a solitary HCC smaller than 5 cm, arguing that the mismatch region in DWI sequences could indicate abnormal microcirculatory perfusion in the liver tissue surrounding the tumor due to microvascular tumor thrombus. Nevertheless, the sensitivity of this feature is limited, and the imaging quality of DWI sequences is prone to interference from image artifacts, necessitating further investigation and validation of its applicability. Previous research has revealed a nonsmooth margin and intratumoral artery as imaging features associated with MVI.^7-10,18,19,25^ MVI typically occurs in liver tissue emerging from irregular cancerous nodules,^25^ whereas intratumoral artery and gene expression variants related to cell proliferation have been linked to stromal invasion.^26^ The current investigation revealed that the AFP levels were not a significant predictor in the nomogram, a deviation from previous reports.^6,7,9,10^ This discrepancy can be attributed to the upper limit of AFP measurements in this particular study group, which was capped at 1210 μg/L, restricting its utility in MVI prediction.

In this investigation, 80.1% of patients with HCC who underwent transarterial cTACE were classified as BCLC Stage A or B. cTACE is the established therapy for individuals with BCLC stage B and offers a viable option for individuals in stage 0 or A who are ineligible for ablation or hepatectomy due to considerations such as age, comorbidities, or tumor location.^11,12^ As a result, incorporating these patients in the study contributes to a more accurate depiction of real-world clinical practice. Superselective cTACE was conducted in this study to the extent of technical feasibility. The amount of pirarubicin and iodized oil administered, as well as the size of gelatin sponge particles used, were contingent upon factors such as tumor size, number, location, tumor-feeding artery vascularity, and liver function of the patient. The main reason for early recurrence after HCC therapy is the micrometastases from the tumor invasion. MVI is a key factor that elevates the chance of early recurrence after radical hepatectomy.^4,5,27-29^ A comparative analysis of tumor progression patterns in 2 patient cohorts stratified as high and low risk for MVI revealed a notable disparity in the incidence of new intrahepatic distant lesions (19.7% vs 32.0%), potentially attributed to the emergence of new HCC lesions within the context of cirrhosis. Conversely, the aggregate frequencies of local recurrence or progression, multiple intrahepatic metastases, and extrahepatic metastases were found to be greater in the high-risk MVI cohort contrasted with the low-risk MVI cohort (80.3% vs 68.0%), potentially stemming from variances in micrometastases caused by tumor infiltration.

Patients categorized as high risk for MVI exhibited a lower first CR rate following conventional cTACE than those classified as low risk for MVI (31.7% vs 51.7%). Existing literature has demonstrated that the initial response to TACE is a significant and reliable prognostic factor.^30^ The research conducted by Bao and Li et al^15^ demonstrated that patients with HCC predicted as proliferative had a lower tumor response rate (CR or PR) following cTACE than patients with HCC identified as nonproliferative (47% vs 67%, *P* = .006), and a shorter PFS duration (7.3 vs 12.1 months, *P* = .002). Previous research revealed that HCC exhibiting a tumor necrosis or fibrosis ratio of more than 90% following TACE typically displays homogeneity and high differentiation, with residual tumor cells predominantly characterized by poor differentiation and insensitivity to hypoxia.^22^ Concurrently, patients who do not achieve CR after TACE have higher levels of serum vascular endothelial growth factor and hypoxia-inducible factor-1α (HIF-1α). HIF-1α upregulates the cyclooxygenase-2 (COX-2) protein expression in residual HCC cells in response to the hypoxic microenvironment, thereby promoting the epithelial-mesenchymal transition process associated with the development of MVI. This mechanism may exacerbate the invasive and metastatic potential of HCC following TACE treatment.^22,31^ As such, our study proposed that patients at high risk of MVI with an incomplete response to cTACE should rather have their follow-up frequency increased.

This investigation is subject to some constraints. First, retrospective cohort studies are inherently susceptible to selection bias. Second, the bulk of research included in this investigation focused on a single center, and the predictive model for MVI was not externally validated. Third, the majority of studies focus on hepatitis B virus infection, emphasizing the importance of multicenter, large-sample validation results on HCC associated with other etiologies.

## Conclusion

We established and verified a predictive model for assessing the risk of MVI using preoperative conventional MRI characteristics with a degree of precision. Patients predicted as high risk for MVI and treated with cTACE demonstrated a greater tumor progression risk within a 2-year timeframe, providing valuable insights for improving treatment strategies in a timely approach.

## Supplementary Material

oyae286_suppl_Supplementary_Tables

oyae286_suppl_Supplementary_Figures

## Data Availability

The datasets generated and/or examined throughout the research may be accessible from the corresponding author upon request.
