# Correction to: Impact of microvascular invasion risk on tumor progression of hepatocellular carcinoma after conventional transarterial chemoembolization

**DOI:** 10.1093/oncolo/oyaf050

**Published:** 2025-03-27

This is a correction to: Guanhua Yang, Yuxin Chen, Minglei Wang, Hongfang Wang, Yong Chen, Impact of microvascular invasion risk on tumor progression of hepatocellular carcinoma after conventional transarterial chemoembolization, *The Oncologist*, 2024, oyae286, https://doi.org/10.1093/oncolo/oyae286

In the ‘Patients’ subsection of the ‘Patients and methods’ section, the sentence beginning ‘Patients were classified into a training cohort (TC; January 2016-December 2010)…’ has been corrected to read: ‘Patients were classified into a training cohort (TC; January 2016-December 2019)…’

