# Supplementary material for: Impact of microvascular invasion risk on tumor progression of hepatocellular carcinoma after conventional transarterial chemoembolization
Source: Oncologist. 2024 Oct 30;30(2):oyae286. doi: 10.1093/oncolo/oyae286 (PMC11884753; doi:10.1093/oncolo/oyae286)
Supplement: oyae286_suppl_Supplementary_Tables [file oyae286_suppl_supplementary_tables.docx]

**Supplementary Tables**

**Supplementary Table S1.** MR Imaging Sequences and Parameters

| **Sequence** | **TR** | **TE** | **FOV** | **Matrix** | **Slice thickness**  **(mm)** | **Inversion angle**  **(degrees)** |
| --- | --- | --- | --- | --- | --- | --- |
| FS-T2-weighted | 7500 | 87 | 350×270 | 388×192 | 5 | 90 |
| DWI | 5100 | 55 | 285×380 | 192×154 | 5 | 90 |
| T1-weighted  (in/out of phase) | 160 | 2.5 | 350×270 | 286×190 | 5 | 90 |
| LAVA | 4.5 | 1.7 | 350×270 | 286×190 | 3 | 10 |
| **Abbreviations:** TR: Repetition time; TE: Echo time; FOV: Field of view; FS: fat-suppression, DWI: diffusion-weighted imaging, performed with b values of 0 and 800 sec/mm^2^. | | | | | | |

**Supplementary Table S2.** Baseline characteristics of patients undergoing cTACE

| **Variable** | **All patients (n=171)** | **Tumor progression**  **within 2 years （n=107）** | **No tumor progression**  **within 2 years**  **（n=64）** | ***P* value** |
| --- | --- | --- | --- | --- |
| Age (years)^a^ | 57(49-66) | 56(48-65) | 59(50-69) | 0.198 |
| Sex |  |  |  | 0.071 |
| male | 140(81.9) | 92(86.0) | 48(75.0) |  |
| female | 31(18.1) | 15(14.0) | 16(25.0) |  |
| Hepatitis B virus Infection |  |  |  | 0.133 |
| absent | 28(16.4) | 14(13.1) | 14(21.9) |  |
| present | 143(83.6) | 93(86.9) | 50(78.1) |  |
| Cirrhosis |  |  |  | 0.906 |
| absent | 49(28.7) | 31(29.0) | 18(28.1) |  |
| present | 122(71.3) | 76(71.0) | 46(71.9) |  |
| Child-Pugh |  |  |  | 0.632 |
| A | 144(84.2) | 89(83.2) | 55(85.9) |  |
| B | 27(15.8) | 18(16.8) | 9(14.1) |  |
| BCLC stage |  |  |  | 0.228 |
| 0 | 34(19.9) | 17(15.9) | 17(26.6) |  |
| A | 50(29.2) | 32(29.9) | 18(28.1) |  |
| B | 87(50.9) | 58(54.2) | 29(45.3) |  |
| Serum creatinine (μmol/L) ^a^ | 63.3(55.9-75.0) | 62.8(56.6-75.4) | 64.8(54.7-73.5) | 0.918 |
| Serum total bilirubin (μmol/L) ^a^ | 19.3(13.3-27.4) | 20.0(14.3-27.8) | 16.4(12.6-26.5) | 0.084 |
| Serum albumin (g/L) ^a^ | 37.7(33.8-40.8) | 37.8(33.8-41.1) | 37.6(32.7-40.4) | 0.440 |
| Prothrombin time (s) ^a^ | 13.5(12.1-14.7) | 13.5(12.2-15.1) | 13.3(11.8-14.4) | 0.131 |
| International normalized ratio ^a^ | 1.1(1.0-1.3) | 1.2(1.1-1.3) | 1.1(1.0-1.2) | 0.035 |
| AST(U/L) ^a^ | 33.4(24.0-48.0) | 33.4(22.5-48.4) | 33.4(25.0-46.5) | 0.822 |
| ALT(U/L) ^a^ | 33.1(24.5-44.3) | 32.2(24.5-43.9) | 33.5(23.8-45.2) | 0.980 |
| Neutrophils count (×10^9^/L) ^a^ | 2.4(1.7-3.3) | 2.4(1.6-3.8) | 2.3(1.7-3.1) | 0.372 |
| lymphocyte count (×10^9^/L) ^a^ | 1.4(1.0-1.8) | 1.3(0.8-1.9) | 1.4(1.1-1.9) | 0.100 |
| Platelet count (×10^9^/L) ^a^ | 124.0(76.0-189.0) | 123.0(72.0-185.0) | 124.5(90.0-191.8) | 0.504 |
| AFP(μg /L) ^a^ | 43.3(5.0-406.9) | 71.4(5.4-1210.0) | 28.1(4.4-1210.0) | 0.268 |
| Tumor diameter (cm) ^a^ | 3.6(2.2-6.4) | 5.2(3.2-7.5) | 2.4(1.7-3.4) | ＜0.001 |
| Tumor number |  |  |  | 0.033 |
| 1 | 73(42.7) | 39(36.4) | 34(53.1) |  |
| ≥2 | 98(57.3) | 68(63.6) | 30(46.9) |  |
| **Abbreviations:** cTACE, conventional transarterial chemoembolization; BCLC, Barcelona Clinic Liver Cancer; AST, aspartate aminotransferase; ALT, alanine aminotransferase; AFP, alpha-fetoprotein; MVI, microvascular invasion.  Unless otherwise specified, the data are the number of patients, with percentages in parentheses; a: the data are presented as median, with interquartile range in parentheses. | | | | |

**Supplementary Table S3.** Frequencies of all imaging features

|  | **Liver resection** | | | |  | **cTACE** | |
| --- | --- | --- | --- | --- | --- | --- | --- |
| **Variable** | **All patients**  **(n=258)** | **Training cohort**  **(n=208)** | **Validation cohort**  **(n=50)** | ***P* value** |  | **Application**  **Cohort**  **(n=171)** | ***P* value** |
| Tumor margin |  |  |  | 0.468 |  |  | 0.075 |
| smooth | 94(36.4) | 78(37.5) | 34(68.0) |  |  | 77(45.0) |  |
| non-smooth | 164(63.6) | 130(62.5) | 16(32.0) |  |  | 94(55.0) |  |
| Tumor growth pattern |  |  |  | 0.092 |  |  | 0.003 |
| Intrahepatic growth | 151(58.5) | 127(61.1) | 24(48.0) |  |  | 124(72.5) |  |
| extrahepatic growth | 107(41.5) | 81(38.9) | 26(52.0) |  |  | 47(27.5) |  |
| Intratumoral artery |  |  |  | 0.959 |  |  | 0.027 |
| absent | 135(52.3) | 109(52.4) | 26(52.0) |  |  | 108(63.2) |  |
| present | 123(47.7) | 99(47.6) | 24(48.0) |  |  | 63(36.8) |  |
| Peritumoral enhancement |  |  |  | 0.475 |  |  | 0.428 |
| absent | 181(70.2) | 148(71.2) | 33(66.0) |  |  | 126(73.7) |  |
| present | 77(29.8) | 60(28.8) | 17(34.0) |  |  | 45(26.3) |  |
| Tumor capsule |  |  |  | 0.711 |  |  | 0.396 |
| complete | 104(40.3) | 85(40.9) | 19(38.0) |  |  | 76(44.4) |  |
| incomplete or absent | 154(59.7) | 123(59.1) | 31(62.0) |  |  | 95(55.6) |  |
| Mosaic architecture |  |  |  | 0.569 |  |  | ＜0.001 |
| absent | 102(39.5) | 84(40.4) | 18(36.0) |  |  | 104(60.8) |  |
| present | 156(60.5) | 124(59.6) | 32(64.0) |  |  | 67(39.2) |  |
| Intratumoral hemorrhage |  |  |  | 0.156 |  |  | ＜0.001 |
| absent | 200(77.5) | 165(79.3) | 35(70.0) |  |  | 155(90.6) |  |
| present | 58(22.5) | 43(20.7) | 15(30.0) |  |  | 16(9.4) |  |
| Intratumoral necrosis |  |  |  | 0.803 |  |  | 0.001 |
| absent | 169(65.5) | 137(65.9) | 32(64.0) |  |  | 138(80.7) |  |
| present | 89(34.5) | 71(34.1) | 18(36.0) |  |  | 33(19.3) |  |
| DWI/T2WI mismatch |  |  |  | 0.076 |  |  | 0.042 |
| absent | 186(71.1) | 155(74.5) | 31(62.0) |  |  | 138(80.7) |  |
| present | 72(27.9) | 53(25.5) | 19(38.0) |  |  | 33(19.3) |  |
| Typical enhancement |  |  |  | 0.157 |  |  | 0.287 |
| absent | 61(23.6) | 53(25.5) | 8(16.0) |  |  | 33(19.3) |  |
| present | 197(76.4) | 155(74.5) | 42(84.0) |  |  | 138(80.7) |  |
| **Abbreviations:** cTACE, conventional transarterial chemoembolization.  Unless otherwise specified, the data are the number of patients, with percentages in parentheses. | | | | | | | |

**Supplementary Table S4.** Baseline characteristics of Liver resection and cTACE groups before and after propensity score analysis

|  | **Before Propensity Score Matching** | | | |  | **After Propensity Score Matching** | | | |
| --- | --- | --- | --- | --- | --- | --- | --- | --- | --- |
| **Matching variable** | **Liver resection**  **(n= 208)** | **cTACE**  **(n = 171)** | ***P* value** | **SMD** |  | **Liver resection**  **(n = 145)** | **cTACE**  **(n = 145)** | ***P v*alue** | **SMD** |
| Age (years) ^a^ | 55(48-65) | 57(49-66) | 0.079 | 0.197 |  | 57(48-65) | 56(48-65) | 0.896 | 0.068 |
| Sex (male) | 159(76.4) | 140(81.9) | 0.197 | 0.134 |  | 113(77.9) | 119(82.0) | 0.378 | 0.023 |
| Hepatitis B virus Infection | 181(87.0) | 143(83.6) | 0.351 | 0.096 |  | 123(84.8) | 125(86.2) | 0.739 | 0.053 |
| Cirrhosis | 148 (71.2) | 122 (71.3) | 0.967 | 0.004 |  | 101(69.7) | 103(71.0) | 0.797 | 0.042 |
| Child-Pugh |  |  | 0.033 | 0.219 |  |  |  | 0.342 | 0.031 |
| A | 190(91.3) | 144(84.2) |  |  |  | 127(87.6) | 132(91.0) |  |  |
| B | 18(8.7) | 27(15.8) |  |  |  | 18(12.4) | 13(9.0) |  |  |
| Tumor diameter (cm) ^a^ | 4.9(2.9-7.8) | 3.6(2.2-6.4) | 0.002 | 0.303 |  | 4.4(2.7-7.0) | 4.0(2.5-6.8) | 0.411 | 0.069 |
| **Abbreviations:** cTACE, conventional transarterial chemoembolization.  Unless otherwise specified, the data are the number of patients, with percentages in parentheses; ^a^: the data are presented as median, with interquartile range in parentheses.  SMD was defined as follows: <0.1, very small differences; 0.1-0.3, small differences; 0.3-0.5, moderate differences; and >0.5, large differences. The matched groups’ comparison was performed with the Mann–Whitney U test for continuous variables and the McNemar test or McNemar-Bowker test for categorical variables, as appropriate. | | | | | | | | | |
