# Supplementary material for: Impact of microvascular invasion risk on tumor progression of hepatocellular carcinoma after conventional transarterial chemoembolization
Source: Oncologist. 2024 Oct 30;30(2):oyae286. doi: 10.1093/oncolo/oyae286 (PMC11884753; doi:10.1093/oncolo/oyae286)
Supplement: oyae286_suppl_Supplementary_Figures [file oyae286_suppl_supplementary_figures.docx]

**Supplementary Figures**

**
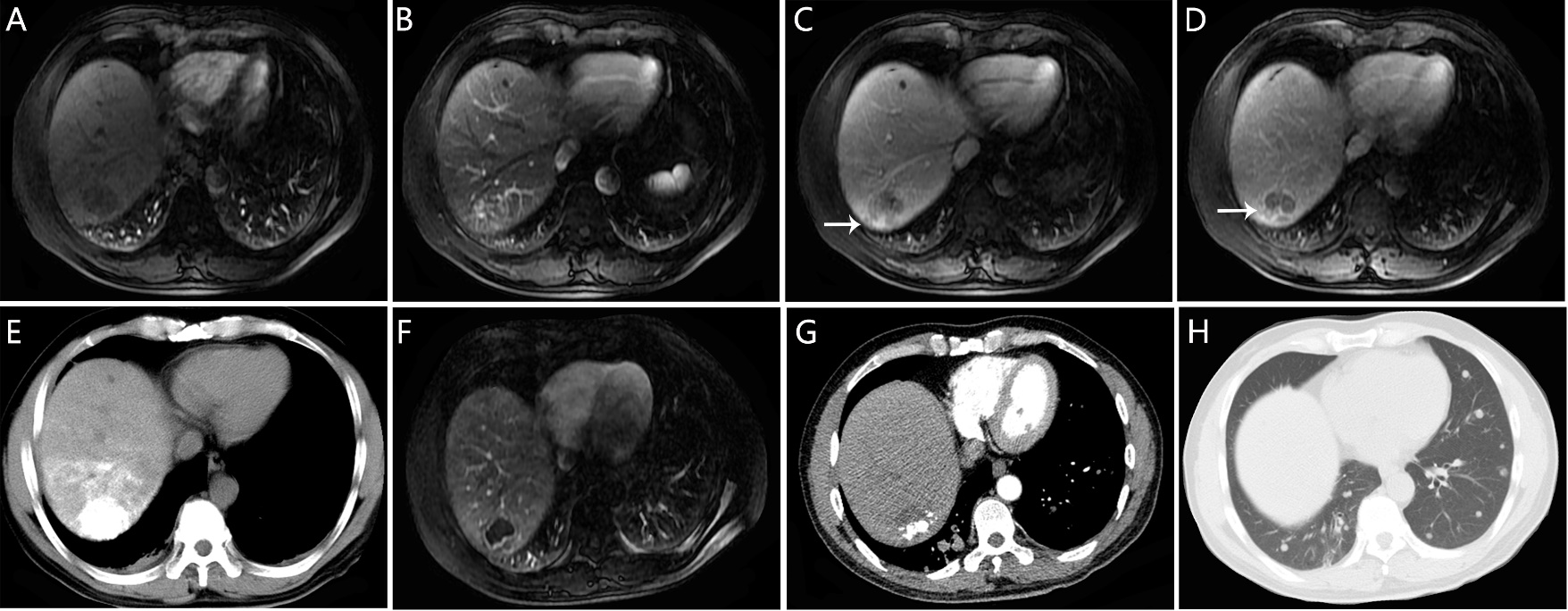
**

**Supplementary Figure 1.** MRI images of a representative case with a high risk of microvascular invasion (MVI). A 54-year-old male patient with a 3.5 cm hepatocellular carcinoma (HCC) located in segment VII. The lesion exhibited low signal intensity on T1-weighted images (A), marked enhancement in the arterial phase on contrast-enhanced scans (B), non-smooth margin in the portal venous phase (C, arrowheads), and an incomplete tumor capsule in the delayed phase (D, arrowheads). The patient achieved a final score of 138 points (38+100), indicating a probability of predicting an MVI greater than 70%. A follow-up examination conducted 3 days post-cTACE revealed satisfactory lipiodol deposition within lesion (E). Subsequent reevaluation 2 months post-TACE demonstrated radiographic complete response in the lesion (F). At reexamination conducted 14 months post-TACE indicated a loss of lipiodol in the lesion (G) and the presence of multiple metastases in the bilateral lungs (H).


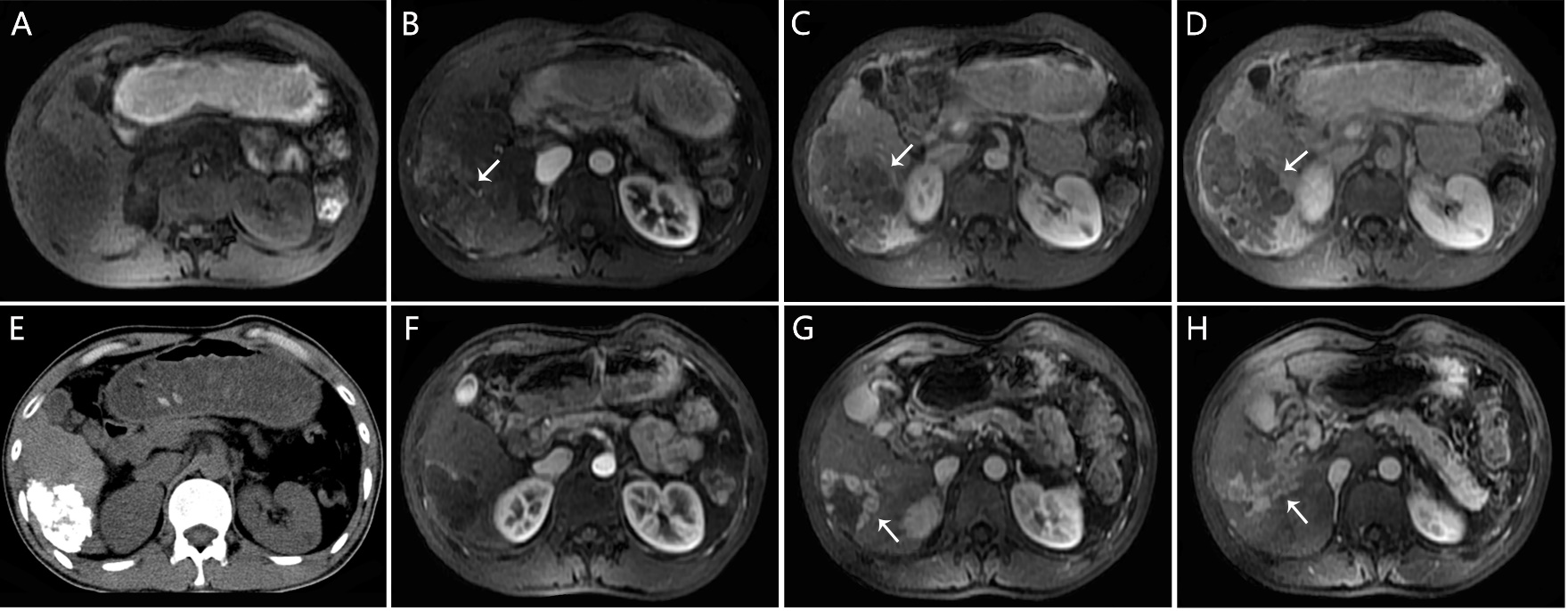


**Supplementary Figure 2.** MRI images of a representative case with a high risk of microvascular invasion (MVI). A 36-year-old male patient with a 10.9 cm hepatocellular carcinoma (HCC) located in segment VI. The lesion exhibited low signal intensity on T1-weighted images (A), intratumoral artery in the arterial phase (B, arrowheads), non-smooth margin in the portal venous phase (C, arrowheads), and an incomplete tumor capsule in the delayed phase (D, arrowheads). The patient achieved a final score of 194 points (56+38+100), indicating a probability of predicting an MVI greater than 90%. A follow-up examination conducted 2 months post-cTACE revealed a reduction in lesion volume and satisfactory lipiodol deposition (E), resulting in radiographic partial response (F). Subsequent reevaluation at 9 months post-cTACE demonstrated local tumor progression (G, arrowheads) and the development of a tumor thrombus in the right portal vein (H, arrowheads).
